# Wnt5A Signaling Promotes Defense Against Bacterial Pathogens by Activating a Host Autophagy Circuit

**DOI:** 10.3389/fimmu.2018.00679

**Published:** 2018-04-09

**Authors:** Suborno Jati, Suman Kundu, Arijit Chakraborty, Sushil Kumar Mahata, Victor Nizet, Malini Sen

**Affiliations:** ^1^Division of Cancer Biology and Inflammatory Disorder, CSIR-Indian Institute of Chemical Biology, Kolkata, India; ^2^Department of Medicine, VA San Diego Healthcare System and University of California, San Diego, La Jolla, CA, United States; ^3^Department of Pediatrics and Skaggs School of Pharmacy and Pharmaceutical Sciences, University of California, San Diego, La Jolla, CA, United States

**Keywords:** pathogen, COPD, sepsis, macrophage, bacterial clearance, autophagy, Wnt5A, actin

## Abstract

Bacterial pathogens are associated with severe infections (e.g., sepsis) and exacerbation of debilitating conditions such as chronic obstructive pulmonary disease (COPD). The interactions of bacterial pathogens with macrophages, a key component of innate immunity and host defense, are not clearly understood and continue to be intensively studied. Having previously demonstrated a role of Wnt5A signaling in phagocytosis, we proceeded to decipher the connection of Wnt5A signaling with infection by pathogenic bacteria, namely *Pseudomonas aeruginosa* (PA) and *Streptococcus pneumoniae* (SP), which are related with the progression of COPD and sepsis. We found that during the initial hours of infection with PA and SP, there is decrease in the steady state levels of the Wnt5A protein in macrophages. Suppression of Wnt5A signaling, moreover, impairs macrophage clearance of the bacterial infection both *in vitro* and *in vivo*. Activation of Wnt5A signaling, on the other hand, enhances clearance of the infection. Macrophage-mediated containment of bacterial infection in our study is dependant on Wnt5A-induced Rac1/Disheveled activation and cytochalasin D inhibitable actin assembly, which is associated with ULK1 kinase activity and LC3BII accumulation. Our experimental findings are consistent with Wnt5A signaling-dependent induction of autophagic killing (xenophagy) of PA and SP, as further substantiated by transmission electron microscopy. Overall, our study unveils the prevalence of a Wnt5A—Rac1—Disheveled-mediated actin-associated autophagy circuit as an important component of innate immunity in host macrophages that may turn out crucial for restricting infection by leading bacterial pathogens.

## Introduction

Macrophages possess an intrinsic ability to confront diverse microbial pathogens and leverage their cytoskeletal dynamics to help maintain the integrity of innate immunity and host defense (1). Yet, several pathogenic microorganisms have evolved into adapting to the intracellular milieu of macrophages for their own benefit (2–5). Leading human pathogens associated with pneumonia, chronic obstructive pulmonary disease (COPD), and sepsis, such as *Pseudomonas aeruginosa* (PA) and *Streptococcus pneumoniae* (SP) are able to defy macrophage clearance in vulnerable hosts, and remain major threats to global health (6). However, despite substantial ongoing research many features of the tussle between the host and the invading pathogens during the key stages of intracellular infection remain unclear at the molecular level. In pursuit of a deeper understanding of host factors orchestrating macrophage-mediated defense against bacterial pathogens, we focused on Wnt5A signaling, which is increasingly recognized to play a role in regulating innate immune and inflammatory responses (7, 8). Previously we had reported that Wnt5A signaling promotes phagocytic uptake but not killing of the non-pathogenic lab strain *E. coli* DH5α (7). In view of the fact that macrophage interactions with bacteria can potentially vary with differences in species and their pathogenicity (2), we investigated the interrelation between Wnt5A signaling and infection by leading bacterial pathogens, such as PA and SP.

Wnt5A belongs to a large family of secreted Wnt glycoprotein ligands, which bind to Frizzled/ROR receptors while participating in various phases of tissue and organ development, morphogenesis, and homeostasis (9–14). In the classical sense, Wnt5A signaling is exemplary of the “non-canonical” mode of Wnt signaling that can operate independently of the transcriptional co-activator β-catenin (13). Frizzled-5, Frizzled-2, and ROR1 are putative receptors for Wnt5A (9, 11, 14). A more intensively studied mode of Wnt signaling, which is by definition β-catenin dependent, is commonly referred to as the “canonical mode” (10, 15). In practice, however, overlap between the signaling intermediates of the canonical and non-canonical modes of Wnt signaling is not infrequent. Decision points between the two modes of Wnt signaling are determined by the availability of Wnt receptors for interaction with the Wnt ligands (16).

Recent studies by our group and others have attributed host cell cytoskeletal motility to Wnt5A signaling (7, 17, 18). Cytoskeletal motility and alterations contribute to phagocytosis and immune homeostasis that strongly influence the initiation and outcome (clearance vs. persistence) of microbial infections (7, 8, 19). Furthermore, Wnt5A signaling plays a pivotal role in macrophage phagocytosis and sustains immune homeostasis in these cells through a Rac1-NFκB-mediated circuitry (8). We thus reasoned that examining the influence of Wnt5A signaling on the sustenance vs. clearance of bacterial infection by macrophages would enhance our understanding of the molecular mechanism of host pathogen interactions, and perhaps reveal key cellular defense strategies that afford protection against virulent or antibiotic-resistant strains.

The cellular microbiology of bacteria-macrophage encounters varies significantly depending on the particular species and strain of the invading organism (2). Attachment and uptake of the bacteria into the host macrophage is followed by microbial interactions with components of the host intracellular milieu, ultimately resulting in either eradication or modulation of infection (host resistance), or establishment of a pathogenic condition (host susceptibility). We undertook this study to understand if Wnt5A signaling regulates host susceptibility vs. resistance to infection caused by important human pathogenic bacteria and whether Wnt5A itself, is affected by the load of bacterial infection. Furthermore, we explored how cellular mechanisms of macrophage-bacterial pathogen interaction are regulated in the context of Wnt5A signaling. To experimentally address our objectives, we manipulated macrophage Wnt5A signaling by exogenous administration of Wnt5A as well as by application of a small molecule inhibitor of Wnt5A production. Our *in vitro* findings were validated *in vivo* through corresponding application of the pharmacological inhibitor and bacterial challenge of both wild-type and Wnt5A-deficient (heterozygous knockout) mice.

Emerging literature highlights how canonical Wnt signaling influences the regulation of immune response mostly through reprogramming of cytokine expression by T cells and dendritic cells (20). Here, we describe how Wnt5A, a representative non-canonical Wnt programs macrophages for host defense against infections by leading bacterial pathogens utilizing host cytoskeleton and the associated Rac1—Disheveled coupled autophagy circuit.

## Materials and Methods

### Cells and Reagents

#### Cell Lines

RAW 264.7 and J774A.1 (murine cell lines), mouse L fibroblast cell line, and mouse L cell line expressing Wnt5A were purchased from ATCC. SP (Strain A-66) were purchased from MTCC, Chandigarh, India. PA strain PA01 and PA14, both clinical isolates were kindly provided by Dr. Victor Nizet, University of California, San Diego and Dr. Chitra Mandal, Indian Institute of Chemical Biology, Kolkata, respectively.

#### Reagents

DMEM–high glucose, RPMI-1640, heat-inactivated FBS, penicillin, streptomycin, l-glutamine, 1× trypsin-EDTA, DAPI (D1306), and phalloidin (A34055) were purchased from Life Technologies. Bacterial agar and medium were purchased from HiMedia Laboratories. Recombinant mouse Wnt5A (645-WN), anti-mouse Wnt5A antibody (AF-645), and anti-human Wnt5A antibody (MAB-645) were purchased from R&D Systems. Anti-β-actin (SC-47778), -Rac1 (SC-217), -LAMP1 (SC-20011) antibodies, and IWP-2 were purchased from Santacruz Biotechnology. Anti LC3B (L7543) antibody, anti-rabbit IgG-HRP conjugate, anti-mouse IgG-HRP conjugate, anti-goat IgG-HRP conjugate, wortmannin (W1628), 3-MA (M9281), cytochalasin D (CytD) (C8273), bafilomycin A1 (B1793), l-α-phosphatidylcholine, and octadecylamine were purchased from Sigma. Anti-Dvl2 (3216S), -Ulk1 (8054S), and -ATG5 (12994) antibodies were purchased from Cell Signaling Technology. Anti p62 (BB-AB0130) antibody, ATP, and cDNA synthesis kit were purchased from Bio-Bharati Life Sciences, Kolkata. Anti Fz5 antiserum raised in rabbit was generated by Bio-Bharati Life Sciences. Soluble 3,3′,5,5′-tetramethylbenzidine (TMB), dithiothrietol, and sucrose were purchased from Calbiochem. PVDF membrane and Luminata classico chemiluminescent substrate for immunoblotting were purchased from Millipore. Rac1 inhibitor (Rac1i) (NSC23766) and Disheveled inhibitor (CAS 294891-81-9) were purchased from Calbiochem. Ulk1 Kinase inhibitor (Ulk1i) (SBI0206965) was purchased from Xcessbio Biosciences Inc. RNA Iso Plus was purchased from Takara. Anti-rabbit Alexafluor 488 (AB150077) was purchased from Abcam.

### Bacterial Uptake and Killing

To assess uptake and total killing of different bacteria, RAW 264.7 cells pretreated with recombinant Wnt5A (50 ng/ml, dissolved in PBS with 0.1% BSA) for 6 h were infected with different bacteria at a multiplicity of infection (MOI) of 10 for different time points from 15 to 120 min at 37°C, 5% CO_2_ in RPMI 1640 without antibiotic. Infected cells were washed and subsequently lysed by sterile distilled water overnight at 4°C. Serial dilution was prepared and diluted aliquots were then spread on agar plates. Colony forming units (CFUs) were counted after incubating the plates overnight at 37°C. As control experiments, PBS with 0.1% BSA was used instead of recombinant Wnt5A (7).

For intracellular killing assays, rWnt5A vs. PBS pretreated RAW 264.7/J774A.1/mouse peritoneal macrophage (PM) cells were infected for 1 h with different bacteria in RPMI/DMEM incomplete medium (without antibiotic) at an MOI of 10, followed by washing with cold PBS to remove extracellular bacteria. Incubation was continued in RPMI/DMEM (with antibiotic; 100 U penicillin/ml and 100 µg streptomycin/ml) for different time-points. Cells were washed and similar procedures followed for CFU calculation. Killing percentage of bacteria was calculated by following equation: (*Initial CFU* − *Final CFU*/*Initial CFU*) × *100*. For checking effect of inhibitor of Wnt production (IWP2), macrophage cells were infected for 1 h with different bacteria in RPMI medium, washed to remove extracellular bacteria subsequent to which cells were treated with either 0.05 µM IWP-2 or DMSO for 24 h in RPMI (with antibiotic) and processed similarly for CFU enumeration to assess killing.

For estimating inhibition by designated inhibitors, specific concentrations as noted in the figure legends were added for the last 3 h of Wnt5A or PBS incubation before infection with bacteria. Similar to other killing assays, infection time was 1 h followed by washing. Killing was assessed after 3 h postinfection.

For CytD associated killing experiments, RAW 264.7 cells growing in RPMI were pretreated with Wnt5A/PBS for 6 h and bacterial infection was given at MOI: 10 for 1 h. Cells were then washed extensively with PBS and incubation in RPMI continued in presence of antibiotic and CytD (1.25 µM) or DMSO (control). Killing was assessed after 3 h postinfection.

Genomic DNA isolation and sequencing of 16S rDNA of clinical isolate of PA was carried out by Eurofins Genomics India Pvt. Ltd.

### Bacterial Infection Models

#### PA Peritonitis

Liposomal formulation of IWP2 (LI) was administered intraperitoneally in 8–12 weeks BALB/c mice and empty liposome was administered as vehicle control. 2 h after LI administration PA infection (CFU 10^6^) was given intraperitoneally. Infected animals were sacrificed after 2 h following which peritoneal cells were isolated and lysed in sterile distilled water for CFU enumeration. Peritoneal lavage supernatant was processed for ELISA.

*Pseudomonas aeruginosa* peritonitis was separately induced in Wnt5A^+/−^ and Wnt5A^+/+^ mice. Mice were sacrificed after 2 h of infection. CFU was estimated by the procedure explained above. Wnt5A level was estimated in peritoneal lavage by ELISA. For establishing Wnt5A^+/−^ and Wnt5A^+/+^ colonies, B6; 129S7-*Wnt5A^tm1Amc^*/J mice were purchased from Jackson Laboratory, USA and bred in in-house animal facility.

#### PA Airway Infection

LI was administered intravenously to 8–12 weeks BALB/c mice and empty liposome was administered as vehicle control. Intranasal inoculation was performed according to published protocol (21). 24 h after LI administration PA (CFU 10^6^) was inoculated intra-nasally and animals were kept for 5 h before sacrifice following which lungs were excised. Whole lung homogenate was made in PBS and lysed in sterile distilled water for CFU measurement. Mice serum was collected for immunoblotting.

#### Sepsis Induction by Cecal Ligation and Puncture (CLP)

CLP was performed according to published protocol (22). Briefly, mice were anesthetized by intraperitoneal injection of ketamine (80–100 mg/kg) and xylazine (5–15 mg/kg). Subsequently, 1–2 cm incision was made aseptically in the lower left abdomen. Then cecum was exposed, ligated at the distal portion, and punctured with a 21-G needle. The incision was sutured in layers and animals were resuscitated. 12 h following surgery, mice were sacrificed and PMs were isolated and lysed in sterile distilled water for estimation of CFU. Peritoneal lavage and serum were collected for ELISA.

### Preparation of L-Wnt5A and L Conditioned Media

L-Wnt5A and L conditioned media were prepared as described previously (7). Briefly, mouse L fibroblast cells and L fibroblast cells expressing Wnt5A were grown in DMEM (high glucose) supplemented with 0.1% G418 sulfate (in case of L fibroblast cells expressing Wnt5A). Following splitting, cells were grown in DMEM (high glucose) without G418 sulfate for 4 days, after which culture supernatant was collected and filter sterilized by passing through 0.2 µM filter. Cells were grown for three subsequent days and culture supernatants were again collected and filter sterilized. These culture supernatants were mixed and used as conditioned media. L cell conditioned medium was prepared by the same procedure.

### Preparation of Liposome-IWP-2

Liposome-IWP2 was prepared as a lipid mixture of L-α-phosphatidylcholine, octadecylamine, and IWP-2 (300 µg) in 20:2:0.3 ratio (8). Briefly, the mixture was dissolved in 1 ml chloroform and solvent was evaporated in rotary evaporator under low pressure. The remnant was procured from the flask by suspension in 1 ml PBS followed by sonication. Excess free drug was removed by two successive washings in PBS by ultracentrifugation (100,000 × *g*, 30 min, 4°C). Vehicle control was prepared accordingly without IWP-2. Presence of IWP-2 in liposome was checked by thin layer chromatography (TLC), where the solvent phase used was acetone:toluene (8:2).

### RNA Isolation and Reverse Transcription PCR (RT-PCR)

RNA was isolated from cells using RNA Iso Plus. cDNA was prepared from isolated RNA by cDNA synthesis kit as described earlier (8). The following primer pairs were used for RT-PCR on freshly prepared cDNA: GAPDH-5′ACCACAGTCCATGCCATCAC3′ (F); 5′TCCACCACCCTGTTGCTGTA3′ (R); mouse Fz5- 5′CTGGGTGCTCATGCTCAAGTAC3′ (F); 5′CGACAGGGACACTTGCTTGTG3′ (R).

### ELISA

For estimating the level of Wnt5A from peritoneal lavage/culture supernatant/sputum, 100 µl of the corresponding sample was coated onto 96-well plates and probed with the appropriate antibodies. After overnight incubation with primary antibody, wells were washed with 0.1% Tween-20 in PBS, after which bound antibody was detected with the appropriate IgG-HRP and developed with TMB substrate. Subsequently, absorbance was measured at 450 nm in an ELISA reader after addition of stop solution (250 mM HCl).

### Western Blotting

Cells were pelleted by centrifugation (2,000 rpm for 5 min at 4°C) and incubated for lysis in cell lysis buffer (50 mM Tris–Cl pH 8.0, 150 mM NaCl, 1% Triton X-100, 0.5% sodium deoxycholate, 0.1% SDS, 50 mM DTT, 5% glycerol, 5 mM NaF, 2 mM Na_3_VO_4_, 50 mM PMSF, 1 mM EDTA, and Protease Inhibitor Cocktail from Calbiochem) for 20 min followed by centrifugation at 12,000 rpm for 5 min at 4°C. In each lane of SDS-PAGE about 40 µg of protein was loaded and after running the protein was transferred to PVDF membrane followed by 2 h of blocking with 5% BSA and subsequent overnight primary antibody incubation at 4°C. Then appropriate HRP-secondary antibodies were added and incubation continued for 2 h at room temperature, following which chemiluminescence reagent was added. Both Image Lab 5.2.1 and GelQuant were used for calculation of band intensities.

### Confocal Microscopy

#### Propidium Iodide (PI) Staining

Propidium iodide staining was done as described previously (23). For evaluating the effect of Wnt5A on bacterial infection Wnt5A and PBS (control), pretreated RAW 264.7 cells were infected with PA (MOI: 1:20), and at 3 h postinfection (T3), all the cells were fixed with 4% paraformaldehyde for 15 min. Then the cells were stained with 30 µM PI and 0.1% saponin in MOPS/MgCl_2_ buffer (0.1 M MOPS, pH 7.2, 1 mM MgCl_2_) for 15 min at room temperature in the dark. Excess stain was washed with MOPS/MgCl_2_ buffer followed by mounting with 60% glycerol. Cells were visualized under Olympus Fluoview FV10i at 60× objective and 3.4× zoom.

Similar staining procedure was followed for PMs harvested after sacrificing PA challenged Wnt5A^+/−^ and wild type mice as well as BALB/c mice previously administered with either liposomal formulation of IWP2 or empty liposomes. Peritoneal cells were plated in complete RPMI media and kept for 6 h. Then the media was removed and the attached cells (mainly macrophages) were processed for staining as described. Cells were visualized under Olympus Fluoview FV10i at 60× objective and 3.4× zoom. PI stained bacteria (red dots) were counted from five different fields of two different experiments.

#### Phalloidin and DAPI Staining

RAW 264.7 cells were plated on four chambered glass slides. After recombinant Wnt5A and inhibitor treatment, the cells were fixed in 4% paraformaldehyde for 15 min. Phalloidin and DAPI staining were done by Alexa Fluor 555 Phalloidin (1:2,000) and DAPI (1:4,000) in 2.5% BSA dissolved in PBST (0.1% Tween-20) for 15 min followed by 3× PBST wash. The slides were mounted with 60% glycerol and visualized under Olympus Fluoview FV10i at 60× objective and 4× zoom. Fluorescence intensity was measured by ImageJ.

#### LC3B Antibody Staining

Samples were prepared for confocal microscopy as described previously (24). Briefly Wnt5A pretreated RAW 264.7 cells were infected with PA (MOI: 1:20) and after 3 h of killing time all the cells were fixed with 4% paraformaldehyde for 15 min. Blocking was done with 2.5% BSA in PBST for 1 h following which antibody (L7543) was added at 0.625 µg/ml and slides incubated overnight. After primary antibody incubation 3× PBST wash was given and secondary antibody was added at 1:3,000 dilution for 1 h. DAPI staining was done after secondary antibody staining following which 3× PBST wash was given. The slides were mounted with 60% glycerol and visualized under Olympus Fluoview FV10i at 60× objective and 3.1× zoom. LC3B punctae (green dots) were counted from about 10 fields from 3 different experiments.

### *In Vitro* Ulk1 Kinase Assay

*In vitro* Ulk1 kinase assay was done as described previously (25). Briefly, cells were lysed with mild lysis buffer (MLB; 10 mM Tris–Cl pH 7.4, 2 mM MgCl_2_, 150 mM NaCl, 0.5% Triton X-100, 10% glycerol, 1 mM Na_3_VO_4_, 50 mM NaF, and Protease Inhibitor Cocktail from Calbiochem). Ulk1 antibody was pre-incubated with protein G-sepharose for 2 h at 4°C in MLB. For pull down, the cell lysate was added to the immune complex and incubated at 4°C for 4 h. The immunoprecipitated (IP) protein was extensively washed with MLB (twice) followed by a single wash with kinase reaction buffer (KRB; 20 mM HEPES, 20 mM MgCl_2_, 2 mM DTT, 100 µM Na_3_VO_4_). For kinase assay, the IP ULK1 bound beads were incubated in KRB (final volume 20 µl) containing 20 µM ATP, 5 μCi [γ−^32^P] ATP, and 5 µg substrate myelin basic protein (MBP) for 20 min at 30°C. The reaction was terminated by adding SDS sample buffer, boiled at 95°C for 10 min, and subjected to SDS-PAGE. The gel was dried and the image was taken using a phosphorimager.

### Phagosome Isolation and Processing

Phagosome isolation was performed as described previously (26). L5a and L conditioned media (7) pretreated RAW 264.7 cells (6 h treatment) were infected with PA (MOI: 10) for 1 h after which fresh RPMI medium was added and incubation continued for 2 h. Subsequently, cells were harvested and washed with ice cold PBS (twice), resuspended in homogenization buffer (HB; 20 mM HEPES/KOH pH 7.2, 0.5 mM EGTA, 250 mM sucrose) and kept for 5 min in ice. Then cells were again pelleted and resuspended in 2 ml HB (containing Protease Inhibitor Cocktail) without EGTA and lysed in dounce homogenizer (130–140 strokes). After that cell homogenate was cleared by centrifugation at 440 × *g* for 3 min at 4°C and 2.4 ml of 65% sucrose was added to the supernatant to make 39% sucrose concentration for the cell homogenate. This 4.4 ml 39% sucrose solution of cell homogenate was layered on top of a discontinuous sucrose gradient consisting of 1 ml 65% sucrose and 2 ml 55% sucrose. Above the cell homogenate layer 2 ml 32.5% sucrose and 1 ml 10% sucrose were added. Finally, ultracentrifugation was done in SW41 rotor at 100,000 × *g* for 1 h. Phagosome was recovered from interface of 55 and 65% sucrose layer and further concentrated by centrifugation at 18 000 × *g* for 10 min. The phagosome pellet was resuspended in HB. SDS-PAGE 4× sample buffer was directly added to the resuspended phagosome and boiled at 95°C for 10 min for immunoblotting. For CFU measurement autoclaved water was added and the resuspended phagosome was plated on LB Agar plate at different dilutions.

### Electron Microscopy

Electron microscopy was performed as described previously (17). Briefly the *Pseudomonas* infected cells of both Wnt5A and PBS-treated sets were fixed in fixation buffer (2% glutaraldehyde and 0.1 M cacodylate) and post fixed in 1% OsO_4_ in 0.1 M cacodylate for 1 h in ice. The cells were stained with 2% uranyl acetate for 1 h in ice and then dehydrated with grades of alcohol. Ethanol and acetone wash was given to dehydrated cells before embedding in Durcupan. Subsequently, 60 nm sections were cut on a Leica UCT Ultramicrotome and supported on Formvar and 300 mesh copper grids. Tecnai Spirit G2 BioTWIN Transmission Electron Microscope with bottle-mount Eagle 4K (16 Megapixel) camera (both from FEI, Hillsboro, OR, USA) was used for viewing of the grids and subsequent photography.

### Statistical Analysis

Results were analyzed with unpaired Student *t* test using Graph-Pad Prism 6 software. Line diagrams and bar graphs are expressed as mean ± SEM. *p* ≤ 0.05 was considered statistically significant. Significance was annotated as follows: *p ≤ 0.05, **p ≤ 0.005, ***p ≤ 0.0005.

### Ethics Statement

All patient samples were collected with informed consent in compliance with the institutional review boards of the Indian Institute of Chemical Biology and the University of California, San Diego. The Animal Ethics Committee of the Indian Institute of Chemical Biology approved the use of animals in the study.

## Results

### Wnt5A Signaling and Infection Load of Pathogenic Bacteria Are Interrelated

**Figure 1:**
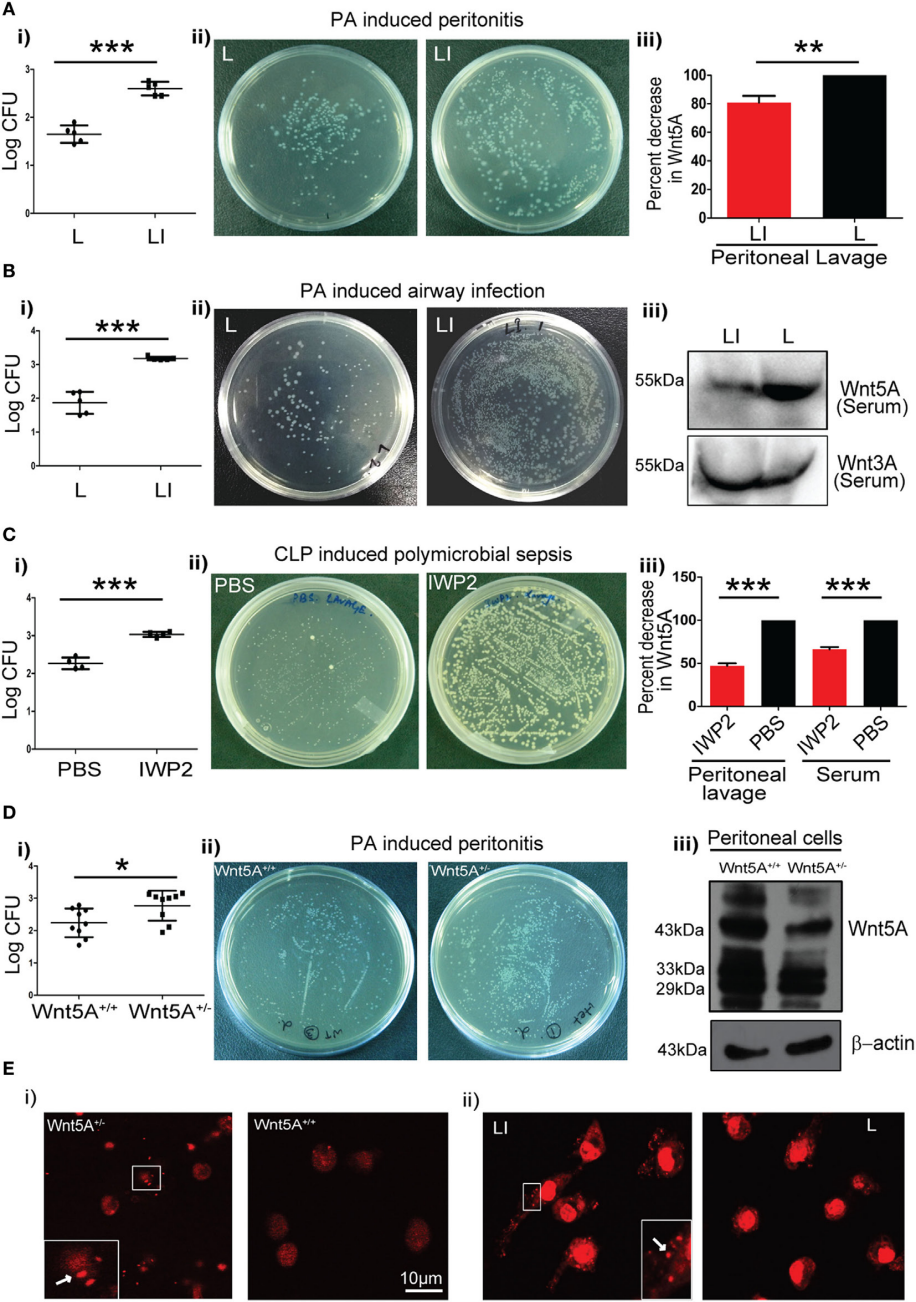
Reduction in Wnt5A level promotes bacterial infection in mouse models. **(A)** Liposomal formulation of inhibitor of Wnt production (IWP2) (LI) and empty liposome (L) were administered intraperitoneally to mice prior to *Pseudomonas aeruginosa* (PA) challenge and mice sacrificed after 2 h (*n* = 5). The difference in bacterial load [colony forming unit (CFU) recovered from peritoneal cells] between the two groups is represented in [**(A)** i,ii]. [**(A)** iii] depicts the inhibitory effect of LI on Wnt5A secretion as estimated from peritoneal lavage. **(B)** LI and L were administered by intravenous route to mice prior to intranasal inoculation with PA and mice sacrificed after 2 h (*n* = 5). The difference in bacterial load is represented in [**(B)** i,ii]. Inhibitory effect of LI on Wnt5A but not Wnt3A secretion after intravenous administration is demonstrated in mice sera by western blotting in [**(B)** iii]. **(C)** IWP2 drug and vehicle control (PBS) were administered as oral gavage for five consecutive days before cecal ligation and puncture (CLP)-induced polymicrobial sepsis and mice sacrificed after 12 h (*n* = 4). [**(C)** i,ii] depict the difference in bacterial load in the peritoneal lavage between the IWP2 and PBS groups. [**(C)** iii] depicts the inhibitory effect of IWP2 on Wnt5A secretion as estimated from peritoneal lavage and serum. **(D)** PA peritonitis was induced in Wnt5A^+/+^ and Wnt5A^+/−^ mice and mice sacrificed after 2 h of infection (*n* = 9). The difference in bacterial load between the two groups is presented in [**(D)** i,ii]. Peritoneal cells of Wnt5A^+/−^ mice express less Wnt5A than the corresponding controls [**(D)** iii]. **(E)** Panels (i,ii) depict the difference in the bacterial load harbored by peritoneal macrophages in LI vs. L and Wnt5A^+/−^ vs. Wnt5A^+/+^ sets of mice. Dots indicated by arrows in insets represent the propidium iodide stained bacteria in the macrophages. Data represented as mean ± SEM; *p ≤ 0.05, **p ≤ 0.005, ***p ≤ 0.0005.

We intended to evaluate the significance of Wnt5A signaling in the context of pathogenic bacterial infections, which are known to be associated with exacerbation of COPD and sepsis (27–31). To this end, we initially utilized two different mouse models of infection: PA peritonitis and PA airway infection. The degree of CLP-induced sepsis in the context of Wnt5A signaling was also noted as a comparison. A small molecule IWP2 was used to block Wnt5A signaling prior to infection, under each condition. Although IWP2, a palmitoleoylation inhibitor may be considered as a general inhibitor of Wnt secretion, the effect of palmitoleoylation inhibition is in fact different for different Wnts (32). For instance, secretion of Wnt5A is affected significantly more than secretion of Wnt3A, and at moderately low doses of IWP2 administration only Wnt5A secretion, not Wnt3A secretion, is affected (7, 8, 32). Specifically, for PA infection studies a liposomal formulation of IWP2 (LI) (~2 mg/kg weight) or empty liposome (L) was administered either intraperitoneally (peritonitis) or intravenously (airway infection). IWP2 (12.5 mg/kg weight) or vehicle control (PBS) was administered as oral gavage for CLP studies. The incorporation of IWP2 into liposome was validated by TLC (Figure S1A in Supplementary Material). The first two panels (i and ii) of Figure 1A (peritonitis model), Figure 1B (airway infection model), and Figure 1C (polymicrobial sepsis model) demonstrate how pharmacological inhibition of Wnt5A secretion by IWP2 intensified bacterial infection, as evidenced by roughly 10-fold increased bacterial CFU harbored in harvested peritoneal cells and lung tissue homogenates. The final panel (iii) confirms the expected reductions in Wnt5A levels in peritoneal lavage and serum achieved by pharmacological blockade with IWP2. In agreement with these studies, peritoneal cells harvested from Wnt5A^+/−^ mice challenged with PA (peritonitis model) exhibited significantly higher bacterial load than those harvested from the Wnt5A^+/+^ (wild type) counterparts 2 h after intraperitoneal infection (Figure 1Di,ii). As expected, peritoneal cells harvested from Wnt5A^+/−^ mice expressed at least twofold less Wnt5A than the wild-type control (Figure 1Diii). Along with other immune cells, macrophages comprise a crucial component of defense that could be modulated by Wnt5A signaling (7, 8, 33, 34). Accordingly, we focused on macrophages to decipher the interrelation between Wnt5A signaling and pathogenic bacterial infection. Indeed, PMs of Wnt5A^+/−^ mice and liposomal IWP2 administered mice challenged with PA contained considerably more PI stained bacteria than those of PA-infected wild type and empty liposome administered counterparts, respectively, as documented by confocal microscopy (Figure 1E; Figures S1B,C in Supplementary Material). These results are clearly indicative of suppressed bacterial clearance by PMs upon Wnt5A depletion.

**Figure 2:**
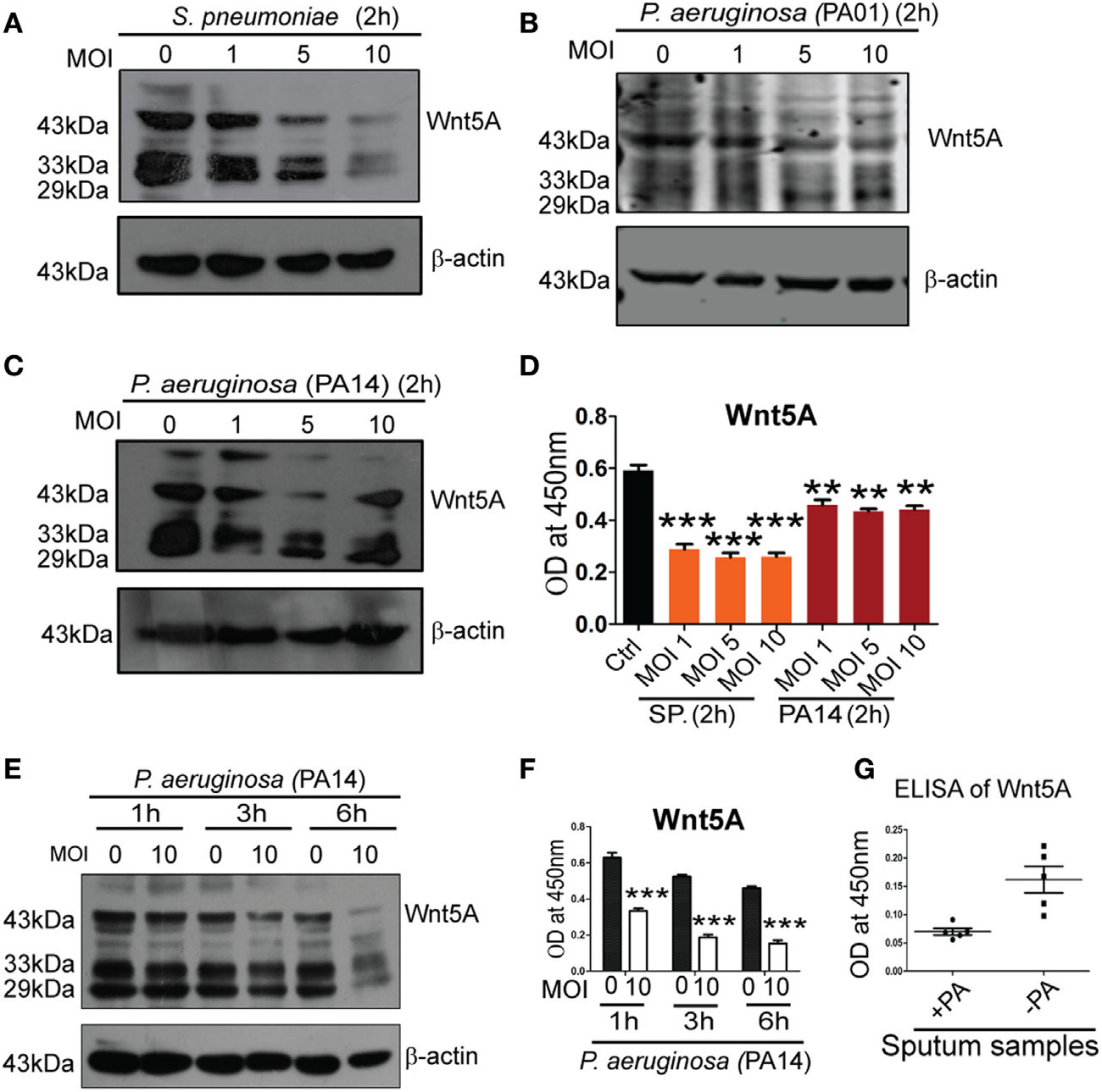
*Pseudomonas aeruginosa* (PA) and *Streptococcus pneumoniae* (SP) infection lead to reduction of both cell associated and secreted Wnt5A. There is decrease in both cell associated **(A–C)** and secreted **(D)** Wnt5A upon infection with PA (strain: PA14 and PA01) and SP (strain: A66) using different multiplicity of infection (MOI) (*n* = 3). MOI 10 infection of PA for different lengths of time produces similar results **(E,F)** (*n* = 3). **(G)** Wnt5A level in sputum samples of COPD patients with and without PA infection (*n* = 5). Data represented as mean ± SEM; *p ≤ 0.05, **p ≤ 0.005, ***p ≤ 0.0005.

To determine how intracellular bacterial infection *per se* affects Wnt5A signaling in macrophages we compared levels of cell-associated and secreted Wnt5A in PA- and SP-infected vs. uninfected macrophages using different multiplicities of infection (MOI: bacteria/cell) and durations of infection. After 2 h infection of RAW 264.7 macrophages with SP and two strains of PA (PA01, PA14) at three different MOI (1, 5, and 10) there was noticeable decrease in both cell-associated Wnt5A (Figure 2A: ~14−80% decrease, Figure 2B: ~20–64% decrease, Figure 2C: ~27–50%) and secreted Wnt5A (Figure 2D: ~34–50% decrease). A similar alteration in cell-associated Wnt5A (Figure 2E) and secreted Wnt5A (Figure 2F) was also obtained after PA infection of RAW 264.7 cells at MOI: 10 with PA, at three different time points (1, 3, and 6 h). Additionally, PA-infected COPD patient sputum samples were found to harbor less Wnt5A than those infected with normal microflora and other bacteria (SP was reported to be absent in each of the COPD sputum samples tested) (Figure 2G). Our results indicate that Wnt5A levels and pathogenic bacterial load are inversely correlated and that macrophage Wnt5A signaling can potentially be suppressed by bacterial pathogens associated with COPD and other important human infections. Other studies demonstrating the relation of Wnt signaling and its intermediates with intracellular bacterial infection have also been reported (35, 36).

### Wnt5A Signaling in Macrophages Facilitates Killing of Pathogenic Bacteria

**Figure 3:**
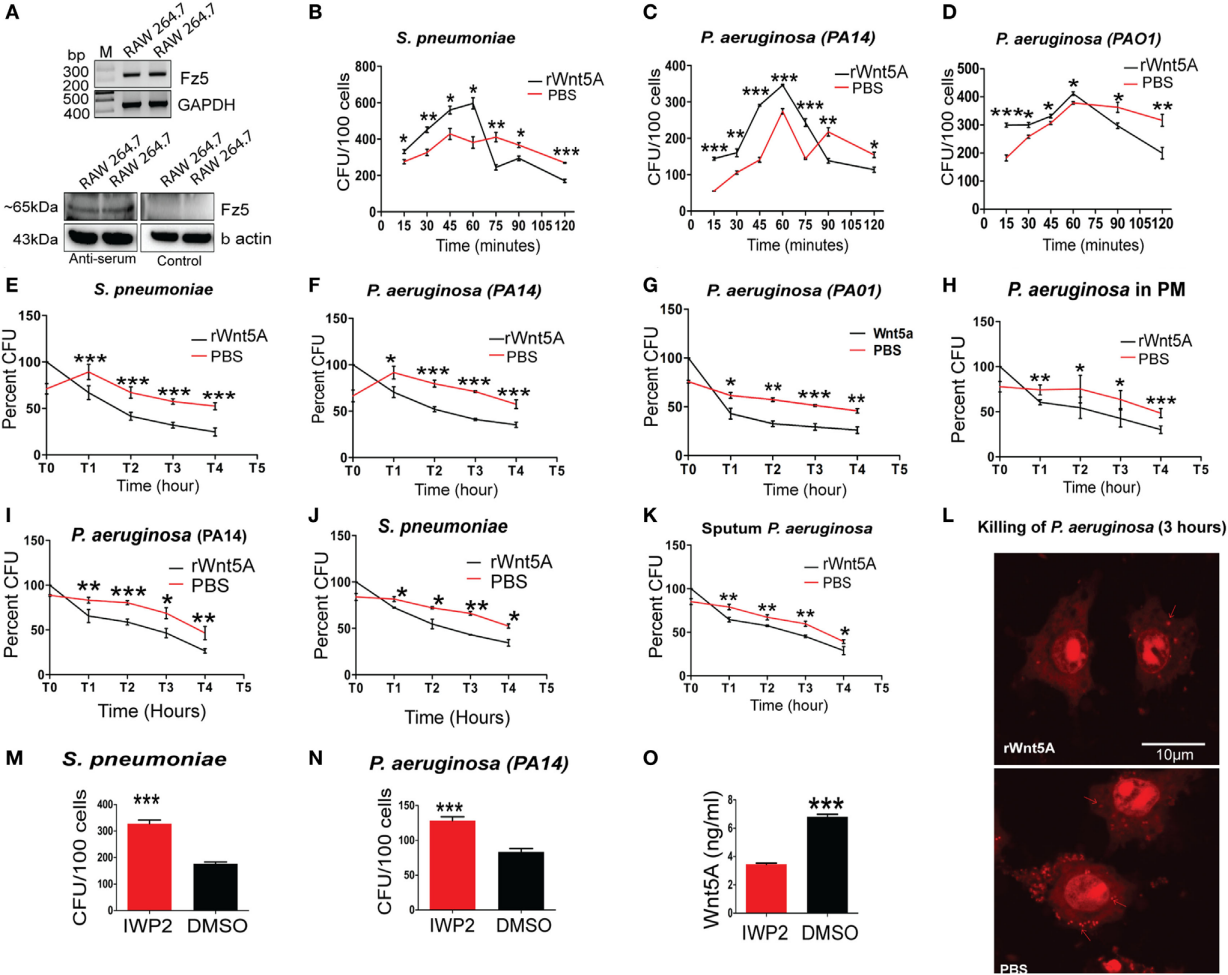
Wnt5A signaling promotes internalization and killing of pathogenic bacteria. **(A)** Reverse transcription PCR analysis (upper panel) and immunoblot (lower panel) demonstrating Fz5 (putative Wnt5A receptor) expression in RAW 264.7 macrophages using anti-Frizzled-5 antiserum (Immune serum) and pre-immune serum (control). **(B–D)** Effect of rWnt5A on internalization and total killing of *Streptococcus pneumoniae* (SP) **(B)**, *Pseudomonas aeruginosa* (PA): PA14 **(C)**, and PA01 **(D)** as estimated by colony forming unit (CFU) (*n* = 3). **(E–G)** Facilitated intracellular killing of SP **(E)**, PA14 **(F)**, and PA01 **(G)** in RAW 264.7 as estimated by percent CFU recovered at different time points (T1–T4) after 1 h infection (T0) (*n* = 3). **(H)** Wnt5A-mediated killing of PA in mouse peritoneal macrophages (*n* = 3). **(I,J)** Wnt5A-induced intracellular killing of PA14 **(I)** and SP **(J)** in J774A.1 at different time points (T1–T4) after 1 h infection (T0) (*n* = 3). **(K)** Similar killing activity of Wnt5A on RAW 264.7 macrophages infected with PA strain isolated from COPD patient sputum (*n* = 3). **(L)** rWnt5A facilitated bacterial killing depicted by confocal microscopy. Red dots indicate propidium iodide stained bacteria 3 h postinfection in RAW 264.7 macrophages pretreated either with rWnt5A or PBS. **(M,N)** Inhibitor of Wnt production (IWP2)-mediated inhibition (24 h of IWP2 treatment after 1 h infection) of Wnt5A production leading to significant sustenance of SP **(M)** and PA **(N)** in RAW 264.7 (*n* = 6). **(O)** ELISA documenting IWP2 mediated reduction in Wnt5A secretion from RAW 264.7 macrophages (*n* = 3). Data represented as mean ± SEM; *p ≤ 0.05, **p ≤ 0.005, ***p ≤ 0.0005.

Our observations that pathogenic bacterial burden was increased in different infection models upon inhibition of Wnt5A signaling (Figure 1) suggested a role for Wnt5A signaling in antibacterial immunity. In earlier work, we demonstrated that Wnt5A signaling promotes phagocytic uptake but not killing of the non-pathogenic *E. coli* lab strain DH5α (7). Here, we undertook a comprehensive analysis of how Wnt5A signaling impacts both internalization and killing of pathogenic bacteria by macrophages. RAW 264.7 murine macrophages pretreated either with recombinant Wnt5A or vehicle control (PBS) were infected separately with the leading pathogens PA and SP, following which both phagocytic uptake of bacteria and their intracellular survival were assessed over time. Figure 3A confirms that all macrophages used for our study expressed the putative Wnt5A receptor Frizzled-5 (8), indicating that they are capable of both autocrine and paracrine modes of Wnt5A signaling. As assessed by recovery of CFU in the initial 2 h, Wnt5A increased the initial uptake of both SP and PA bacterial strains (MOI = 10) (Figures 3B–D). However, thereafter, CFU recovery of both SP and PA dropped more rapidly in Wnt5A-treated macrophages compared to PBS-treated controls indicating greater total killing of the pathogenic bacteria when Wnt5A signaling was activated. To specifically examine intracellular killing of the pathogenic bacteria, both Wnt5A- and PBS-pretreated RAW 264.7 and PMs were infected separately with PA or SP for 1 h, then washed to remove extracellular bacteria and harvested at different time points over a 4 h time course (T1–T4) to assess viable intracellular bacteria through CFU enumeration. Wnt5A accelerated intracellular killing of SP (~50%) and PA (40–50%) in both RAW 264.7 and PMs (Figures 3E–H). Infection of a second macrophage line (J774.1A) pretreated with Wnt5A vs. PBS produced similar results (Figures 3I,J). Wnt5A treatment also facilitated killing of a PA strain isolated from patient sputum by about 35% (Figure 3K). The identity of the isolate was validated by 16SrRNA sequence (Figures S2A,B in Supplementary Material). Wnt5A-mediated enhancement of bacterial killing was corroborated by confocal microscopy using PI stained PA (Figure 3L). To further validate the importance of Wnt5A signaling in controlling internalized bacterial pathogens, macrophages were first infected with PA and SP, and IWP2-mediated inhibition of Wnt5A signaling was continued for 24 h before recovery and enumeration of intracellular CFU. As depicted in Figures 3M,N, IWP2-treated macrophages harbored about 40% more viable bacteria than controls. Decrease in Wnt5A secretion in response to IWP2 was confirmed by ELISA (Figure 3O).

### Wnt5A-Mediated Killing of Pathogenic Bacteria Is Rac1/Disheveled-Dependent Implicating Actin Involvement

**Figure 4:**
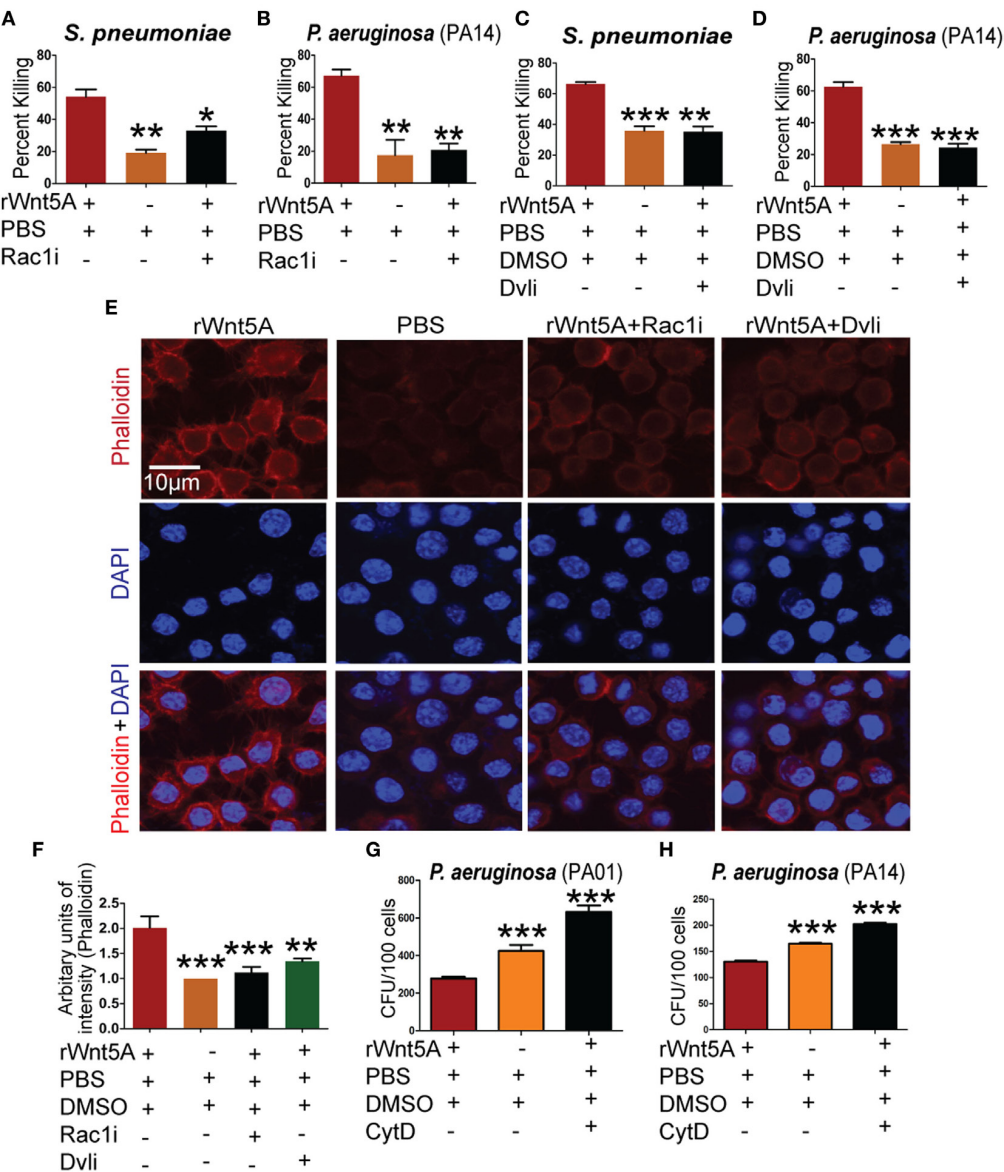
Wnt5A-mediated bacterial killing is Rac1–Dvl dependent and involves actin assembly. rWnt5A-induced clearance of bacteria [*Streptococcus pneumoniae* (SP) and *Pseudomonas aeruginosa* (PA)] was inhibited by Rac1 inhibitor (Rac1i) **(A,B)** and Dvl inhibitor (Dvli) **(C,D)** at 3 h (T3) postinfection (*n* = 3). PBS and DMSO act as vehicle control. **(E)** Inhibitory effect of Rac1i and Dvli on Wnt5A-induced actin polymerization as detected by phalloidin staining. **(F)** Difference in phalloidin fluorescence intensities calculated by NIH-ImageJ software (*n* = 3). **(G,H)** Inhibitory effect of cytochalasin D (CytD) (postinfection) on Wnt5A-mediated bacterial killing. Data represented as mean ± SEM; *p ≤ 0.05, **p ≤ 0.005, ***p ≤ 0.0005.

Rac1 and Disheveled are known intermediates of Wnt5A signaling (37–39) and could play a role in SP and PA killing by macrophages. To examine this possibility, commercially available Rac1 and Disheveled inhibitors (Rac1i and Dvl inhibitor) (7, 40) were used separately at 5 µM concentration in combination with Wnt5A to assess differences in bacterial killing under the same experimental conditions as employed above. Wnt5A-induced killing of PA was reduced significantly in the presence of Rac1 (Figures 4A,B) and Disheveled (Figures 4C,D) inhibitors, implying that both intermediates contribute to Wnt5A-mediated regulation of intracellular bacterial killing. Both inhibitors also reduced the Wnt5A-induced actin assembly in RAW 264.7 macrophages as measured by phalloidin staining (Figure 4E; quantification in Figure 4F).

A number of bacterial pathogens exploit the actin cytoskeleton to establish intracellular infection (19). We note that Wnt5A signaling, which defends against bacterial infection influences actin assembly. Thus, we hypothesized that Rac1 and Disheveled inhibitors could inhibit Wnt5A-mediated bacterial killing by suppressing the effect of Wnt5A on actin assembly. Consistent with this model Wnt5A-induced killing of PA after internalization was blocked by treatment with CytD, an inhibitor of actin assembly (41) (Figures 4G,H).

### Wnt5A Signaling-Mediated Autophagy Facilitates Bacterial Clearance (Xenophagy) in Macrophages

**Figure 5:**
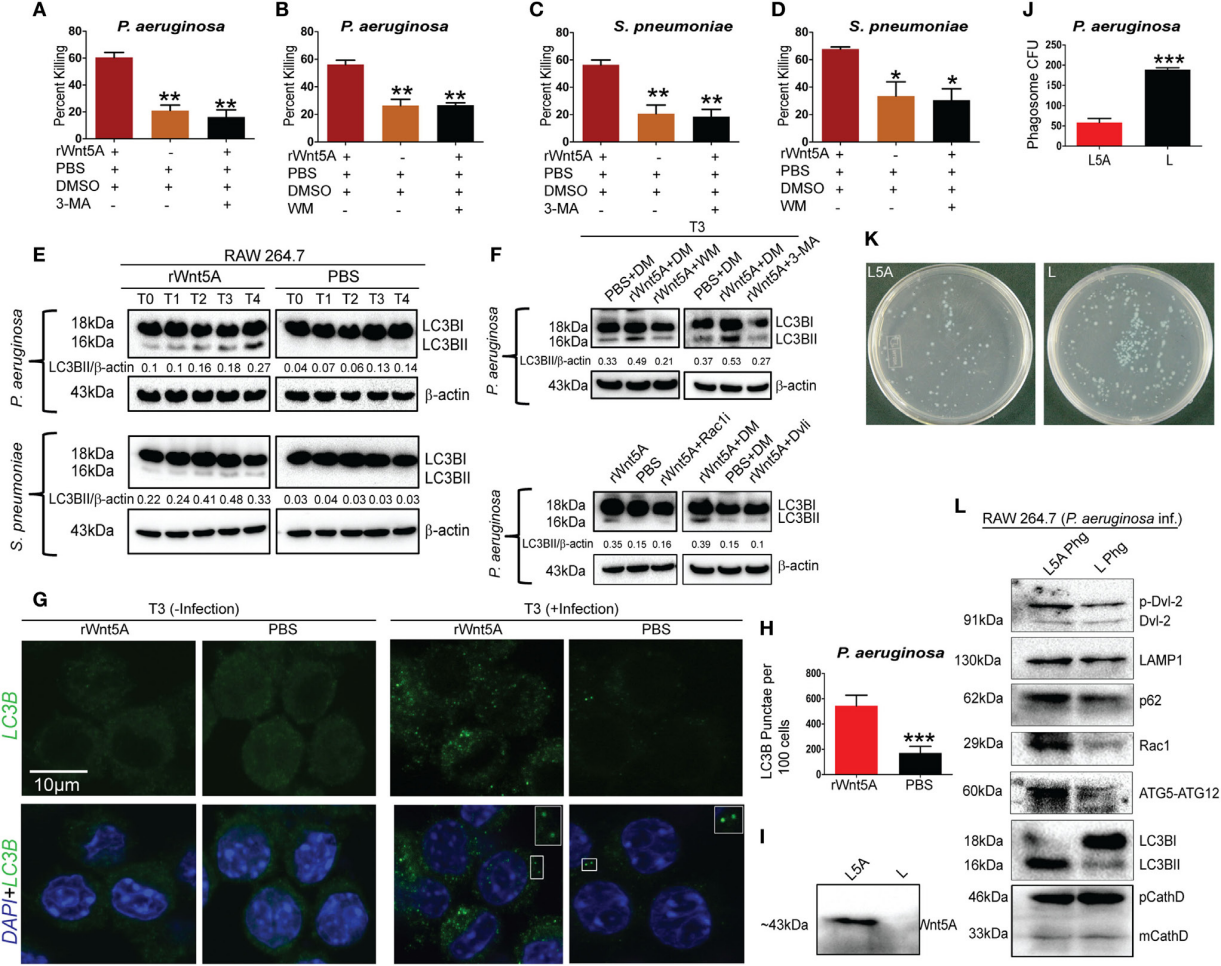
Wnt5A-induced bacterial clearance is mediated through autophagy. **(A–D)** Effect of 3-methyladenine (3-MA, 1 mM) and wortmanin (WM, 100 nM), respectively, on rWnt5A-mediated killing of *Pseudomonas aeruginosa* (PA) and *Streptococcus pneumoniae* (SP) (*n* = 3). Percent killing was calculated for 3 h (T3) killing time-point. DMSO (DM) and PBS were used as vehicle control. **(E)** LC3BII accumulation assessed at different killing time-points (T0–T4) for rWnt5A and PBS pretreated RAW 264.7 cells infected separately with PA and SP by western blotting (*n* = 3). T0 represents bacterial killing in 0 time (post 1 h of infection). **(F)** Influence of WM, 3-MA, Rac1 inhibitor, and Dvl inhibitor (Dvli) on rWnt5A-induced LC3BII accumulation at 3 h (T3) postinfection with PA (*n* = 3). **(G,H)** LC3-punctae in Wnt5A vs. PBS sets as assessed by confocal microscopy **(G)** and punctae dot calculation **(H)** (*n* = 3). **(I)** Presence of Wnt5A in L5A conditioned medium (L5A: L cells expressing Wnt5A) but not in L conditioned medium (used as control) (*n* = 3). **(J,K)** Difference in bacterial load between L5A Phg (phagosome isolated from L5A treated cells 2 h postinfection: T2) and L Phg (corresponding control) (*n* = 3). **(L)** Higher levels of LC3BII, ATG5-ATG12, Rac1, p62, LAMP-1, and p-Dvl-2 (phosphorylated Disheveld-2) in L5A Phg compared to L Phg. Both sets contain similar levels of precursor and mature forms of cathepsin D (CathD). Data represented as mean ± SEM; *p ≤ 0.05, **p ≤ 0.005, ***p ≤ 0.0005.

Given the known association of Wnt5A signaling with cytoskeletal dynamics (18) and the multifaceted roles of the actin cytoskeleton in the initiation of autophagy (42, 43), we investigated the possibility that Wnt5A-mediated bacterial killing involves utilization of host autophagy machinery. The effect of the autophagy inhibitors 3-methyladenine (3-MA, 1 mM) and wortmannin (WM, 100 nM) on Wnt5A-mediated bacterial killing was assessed, based on reports by other investigators (42, 44, 45). Being wary of the off-target effects produced by 3-MA and WM, we used lower concentrations than those reported of these drugs. Killing of both PA (Figures 5A,B) and SP (Figures 5C,D), 3 h postinfection was inhibited, when 3-MA or WM were administered during activation of Wnt5A signaling. Immunoblot analysis of lysates prepared from macrophages infected separately with SP and PA at time points corresponding to increased Wnt5A-mediated bacterial killing revealed significantly increased level (~70–80%) of LC3BII, a well characterized marker of autophagy (44, 45) in Wnt5A-treated vs. PBS-treated cells (Figure 5E). Lesser accumulation of Wnt5A-induced LC3BII in case of SP infection as compared to PA infection is possibly on account of the difference between the two bacterial species in their interactions with the host. The increase in Wnt5A-induced LC3BII levels was significantly reduced (~50–60%) upon administration of either 3-MA or WM (Figure 5F: upper panel), consistent with autophagy as a mechanism of bacterial killing. On the contrary, Wnt5A-induced LC3BII levels increased significantly (~80–90%) upon application of bafilomycin-A1 (25 nM), which inhibits autophagic flux by blocking lysosomal fusion with autophagosomes (46), further supporting the occurrence of autophagy (Figure S3 in Supplementary Material). Moreover, inhibitors of Rac1 and Disheveled decreased Wnt5A-induced accumulation of LC3BII by about 60%, validating involvement of both intracellular effectors of Wnt5A signaling in autophagy-mediated bacterial clearance (Figure 5F: lower panel). Similar results were found in studies of macrophage infection with the Gram-positive bacterial pathogen SP (Figures S3 and S4 in Supplementary Material). Wnt3A, considered as a reference in our studies, did not have significant effect on pathogenic bacterial clearance, when compared to Wnt5A (Figures S5A,B in Supplementary Material). Figures 5G,H show significantly increased LC3BII puncta in PA-infected cells pretreated with Wnt5A at 3 h after infection (T3) in corroboration of the already documented results. To further evaluate Wnt5A-induced autophagy in bacterial clearance we isolated phagosomes from PA-infected macrophages pretreated with either Wnt5A-conditioned medium or control medium using an established phagosome isolation protocol (26). Figure 5I demonstrates presence of Wnt5A in the conditioned medium prepared from L-Wnt5A cells but not L cells. We found significantly reduced bacterial recovery in phagosomes isolated from L-Wnt5A-treated macrophages vs. control (Figures 5J,K). Furthermore, higher levels of LC3BII as well as ATG5-ATG12 were present in the phagosomes isolated from Wnt5A-conditioned medium treated sets as opposed to the control sets (Figure 5L). Considering that the ATG5-ATG12 conjugate is associated with LC3BII incorporation into autophagosomes (47), our observations suggest that the phagosomes from Wnt5A-treated infected macrophages harbor more autophagosome-like features than their control counterparts. The autophagosome-like moieties from Wnt5A sets also possessed higher levels of both Rac1 and phosphorylated Disheveled-2 (Figure 5L) suggesting that Wnt5A signaling intermediates Rac1 and Disheveled contribute to autophagosome formation and maturation in infected macrophages. These results are in agreement with the observed role of Rac1 and Disheveled in Wnt5A-induced bacterial killing in macrophages (Figure 4). Moreover, higher levels of p62 and LAMP1 in the autophagosome-like components of Wnt5A-treated sets imply increased levels of p62-assisted bacterial incorporation into these structures and their facilitated fusion with lysosomes (48, 49). Of note, similar levels of precursor and mature forms of cathepsin D (phagosome marker) were present in both Wnt5A and corresponding control sets.

**Figure 6:**
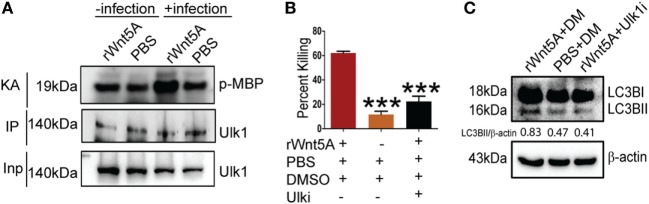
Ulk1 kinase activity (KA) is needed for rWnt5A-mediated bacterial clearance. **(A)** At 2 h killing time point (T2), immunoprecipitated (IP) Ulk1 from rWnt5A-treated RAW 264.7 cells demonstrates greater Ulk1 KA than the corresponding control as judged by phosphorylation of myelin basic protein (MBP). Significant difference in KA not noted in uninfected cells pretreated with Wnt5A or PBS (*n* = 3). Inp (Input) is same in all cases. **(B)** rWnt5A-mediated killing inhibited by Ulk1 kinase inhibitor (Ulk1i) at 3 h (T3) killing time-point (*n* = 3). DM (DMSO) and PBS were used as vehicle control. **(C)** rWnt5A-mediated LC3BII accumulation inhibited in presence of Ulk1i (*n* = 3). Data represented as mean ± SEM; ***p ≤ 0.0005.

ULK1 kinase activity (KA) is required for initiation of autophagy under starvation conditions (25). Thus, we examined if Wnt5A signaling in PA-infected macrophages promoted ULK1 KA. ULK1 was IP from Wnt5A vs. PBS-treated cells infected with PA and the KA of the immunoprecipitate on MBP assessed. As depicted in Figure 6A, Wnt5A-treated cells infected with PA had elevated ULK1 KA as compared to corresponding controls. ULK1 kinase levels were roughly similar in all cases. Administration of Ulk1i (50) at 2.5 µM to Wnt5A-pretreated PA-infected macrophages decreased Wnt5A-mediated killing by 60%, within 3 h (T3) postinfection (Figure 6B). Figure 6C demonstrates that LC3BII accumulation decreased by about 50% in Wnt5A-pretreated PA-infected cells upon administration of the Ulk1i implying that Wnt5A-mediated bacterial killing engages in autophagy utilizing ULK1 kinase activation.

**Figure 7:**
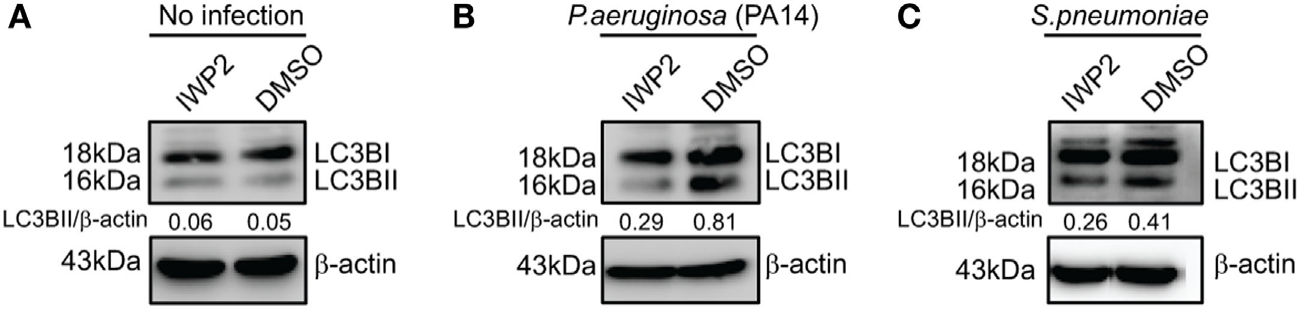
Inhibitor of Wnt production (IWP2) treatment correlates with reduced LC3BII accumulation in infected [*Pseudomonas aeruginosa* (PA), *Streptococcus pneumoniae* (SP)] macrophages. **(A)** In absence of infection, IWP2 does not have significant effect on LC3BII accumulation on RAW 264.7 cells. **(B,C)** Effect of IWP2 treatment on LC3BII accumulation in PA14 **(B)** and SP **(C)** infected macrophages.

The involvement of Wnt5A signaling in autophagic clearance of bacteria was furthermore demonstrated in macrophages, where Wnt5A production was depleted using IWP2. As depicted in Figure 7, SP- and PA-infected RAW 264.7 macrophages (1 h infection) treated with IWP2 for 24 h, accumulated considerably less (40–60%) LC3BII as compared to the corresponding controls, in agreement with suppressed bacterial clearance in Wnt5A depleted condition as depicted before (Figures 3M,N).

**Figure 8:**
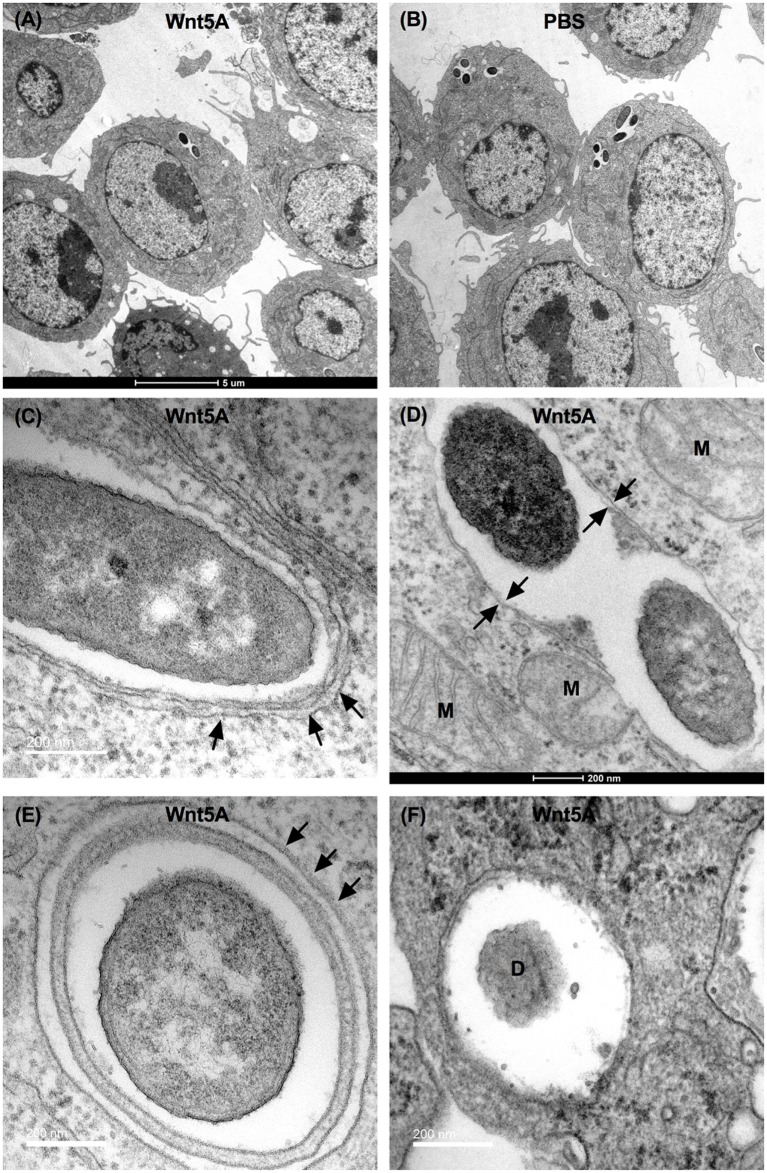
Transmission electron microscopy (TEM) demonstrating Wnt5A-induced autophagosome like moieties and bacterial killing after PA infection. Lesser number of bacteria present in Wnt5A-treated cells compared to PBS-treated cells **(A,B)**. Arrows point to double membrane or multilamellar structures encapsulating bacteria in Wnt5A-treated macrophages **(C–F)**. M represents mitochondria. D denotes degraded bacteria.

Finally, Wnt5A-mediated clearance of pathogenic bacteria through autophagy was substantiated by transmission electron microscopy (TEM). The number of bacteria (PA) per macrophage was much lower in Wnt5A-treated sets than in the corresponding PBS controls (Figures 8A,B). Autophagosome like vesicles possessing double or multi-membranous structures with encapsulated bacteria were prevalent to a considerably greater degree in Wnt5A-treated PA-infected macrophages as compared to the corresponding control (Figures 8C–F; Figure S6 in Supplementary Material). The autophagosome like vesicles in Wnt5A-treated sets also occupied more area than those present in the corresponding controls (Figure S6 in Supplementary Material).

## Discussion

Bacterial infections in vulnerable hosts are a major health problem, especially due to the increasing incidence of antibiotic resistance (6). In this scenario, a clear knowledge of host defense mechanisms against pathogen invasions is of lasting importance. Despite continuous measures to harness the many nuances of host pathogen interactions and the plethora of research articles along this line, several questions still remain in relation to the means of host resistance to pathogen incursions.

Having published an original observation of the involvement of Wnt5A signaling with macrophage phagocytosis using the non-pathogenic laboratory strain *E. coli* DH5α (7), we aimed toward interpreting the connection of Wnt5A signaling with infection by pathogenic bacteria, namely PA and SP, which are related with the progression of COPD and sepsis. This undertaking was driven with the understanding that the molecular dynamics at the interface of bacteria-macrophage interactions may vary significantly depending on the species and strain of the invading organism.

We have found that Wnt5A signaling in the host restricts spread of infection by leading human bacterial pathogens PA and SP, despite their potential to suppress Wnt5a protein levels and macrophages constitute an important component of this innate immune defense program (Figures 1–3). Macrophages promote clearance of PA and SP through Wnt5A—Rac1—Disheveled-dependent actin assembly (Figures 3 and 4), which is associated with the initiation of autophagy (42). Both Wnt5A signaling and bacterial infection act together to stimulate LC3BII accumulation, a well known marker of autophagy (44, 45) and LC3B reactive puncta correlate significantly with Wnt5A facilitated bacterial clearance as depicted by bacterial killing assays, western blotting, and confocal microscopy (Figures 3 and 5). Moreover, activation of Wnt5A signaling in infected macrophages augment the maturation of LC3BII carrying autophagosome like structures that bear the potential to finally execute xenophagic clearance through fusion with lysosomes (xenophagy: killing of bacteria utilizing autophagy machinery). From the raised levels of Rac1 and phosphorylated Disheveled in the autophagosome-like moieties harvested from Wnt5A-treated infected cells, it is clear that Rac1 and Disheveled, Wnt5A signaling intermediates, promote the formation of bacteria-containing autophagosomes (Figure 5). The incidence of a Wnt5A-induced autophagy program in infected macrophages is furthermore corroborated by both increased ULK1 KA in the presence of Wnt5A as well as diminution in bacterial killing and LC3BII accumulation upon administration of an inhibitor of ULK1 kinase (Figure 6). Wnt5A-induced ULK1 KA and Rac1—Disheveled mediated actin modulation pathways possibly synchonize for xenophagy to progress. Additionally, decrease in bacterial killing with Wnt5A depletion correlates with decrease in LC3BII accumulation (Figures 3 and 7) substantiating the need for Wnt5A in autophagy coupled bacterial killing. Taken together, the experimental findings presented here clearly indicate that Wnt5A signaling contributes significantly to xenophagic clearance of bacterial pathogens. Autophagosome-like structures and their further extension with vesicles generated from mitochondria during Wnt5A facilitated autophagosome maturation are in fact evident from TEM (Figure 8; Figure S7 in Supplementary Material). It is not clear at this stage of our study which mode(s) of Wnt5A signaling, non-canonical and/or canonical operate(s) during xenophagy on account of the often encountered overlap between the canonical and non-canonical modes of Wnt signaling (16). Wnt3A, considered as a reference in our studies, does not have significant effect on the pathogenic bacterial clearance (Figure S5 in Supplementary Material).

Our experimental findings indicate that Wnt5A signaling acts toward the containment of infection by both Gram+ *Streptococcus* (SP) and Gram− *Pseudomonas* (PA) strains, implying that Wnt5A—Rac1—Disheveled-dependent cytoskeletal alterations associated with autophagy (xenophagy) initiation may operate independent of the characteristics of different pathogenic bacterial species and strains. However, distinctly separate molecular mechanisms of host pathogen interactions could still be involved in the extent and kinetics of autophagic flux and clearance of the different pathogens after initiation of the host autophagy program. The occurrence of autophagy during infection by *Pseudomonas* species and other bacterial pathogens has been previously reported (51, 52). Interrelation of Wnt signaling pathway intermediates with the host autophagy program during bacterial infection has also been discussed (53). Our study expands on this knowledge through highlighting unidentified host cell signaling pathways involved in coordinating the autophagy circuit during infection by commonly encountered bacterial pathogens.

The means used by different bacterial pathogens to counteract the host autophagy program are diverse. While some directly escape from the phagosome, others exploit autophagosomes for their nutritional needs (52, 54). Whether or how Wnt5A signaling affects progression of infection by such pathogens is an important matter to investigate.

We demonstrate that the impact of Wnt5A signaling on the pathogenic bacteria, PA and SP is different from that on the non-pathogenic lab strain *E. coli* DH5α, where facilitated bacterial killing by Wnt5A signaling is not observed (7). When PA and SP are taken up by macrophage phagocytosis they may attempt to influence host cytoskeletal actin dynamics to promote their own intracellular survival, possibly unlike non-pathogenic lab strains like *E. coli* DH5α. PA and SP initiated cytoskeletal modulation may be antagonized by the intrinsic influence of Wnt5A signaling on actin assembly and thus redirected toward pathogen elimination by autophagic clearance. Additional experiments are needed for a thorough evaluation of this concept, especially in light of a recent article that reports Wnt5A facilitated survival of the bacterial pathogen *Ehrlichia chaffeensis* and pathogenic mycobacteria (55, 56).

The modes of killing of internalized bacteria and those associated with the cell surface may differ. We observed that activation of Wnt5A signaling promoted both intracellular and total bacterial killing of PA and SP upon performing both antibiotic protection as well as total killing assays (Figure 3). Whether Wnt5A-mediated Rac1—Disheveled actin-associated autophagy pathways contribute beyond intracellular bacterial killing is currently not clear.

Wnt5A signaling is known to promote secretion of pro-inflammatory cytokines under pathogenic conditions (57, 58) and the role and outcome of Wnt5A in innate immune defense is likely to be dose and context dependent. While too much Wnt5A signaling and cytokine secretion could be associated with inflammation, depletion or inactivation of Wnt5A signaling may be linked with an immune deficient status, promoting microbial persistence. Further analysis of the magnitude and dynamics of Wnt5A signaling in different experimental contexts and in clinical infectious disease scenarios is thus essential.

Our documentation of regulation of bacterial infections by a Wnt5A—Rac1—Disheveled mediated autophagy circuit unveils an as yet uncharacterized component of host pathogen interactions in the innate immune defense program. This finding may instruct new modes of therapy against drug resistant bacterial pathogens.

## Ethics Statement

All patient samples were collected with informed consent in compliance with the institutional review boards of the Indian Institute of Chemical Biology and the University of California, San Diego. The Animal Ethics Committee of the Indian Institute of Chemical Biology approved the use of animals in the study.

## Author Contributions

MS organized the research. SJ and SK performed research. MS, SJ, and SK analyzed data. MS wrote the paper with assistance from SJ, SK, and VN. SM helped in electron microscopy. AC helped in Wnt5A^+/−^ mouse work. VN helped in bacterial killing and electron microscopy studies, data analysis, and manuscript writing.

## Conflict of Interest Statement

The authors declare that the research was conducted in the absence of any commercial or financial relationships that could be construed as a potential conflict of interest.
