# Supplementary material for: Wnt5A Signaling Promotes Defense Against Bacterial Pathogens by Activating a Host Autophagy Circuit
Source: Front Immunol. 2018 Apr 9;9:679. doi: 10.3389/fimmu.2018.00679 (PMC5900007; doi:10.3389/fimmu.2018.00679)
Supplement: Supplementary file 1 [file Presentation_1.PDF]

Supplementary Material

Wnt5A signaling promotes defense against bacterial pathogens by activating a host autophagy circuit.

Suborno Jati *[, 1], Suman Kundu *[, 1], Arijit Chakraborty [1], Sushil Kumar Mahata [2], Victor Nizet [3] and Malini Sen [1,¶]

[¶]**Corresponding author:** To whom correspondence should be addressed. E- mail: msen648@gmail.com, msen@iicb.res.in

Figure S1.a

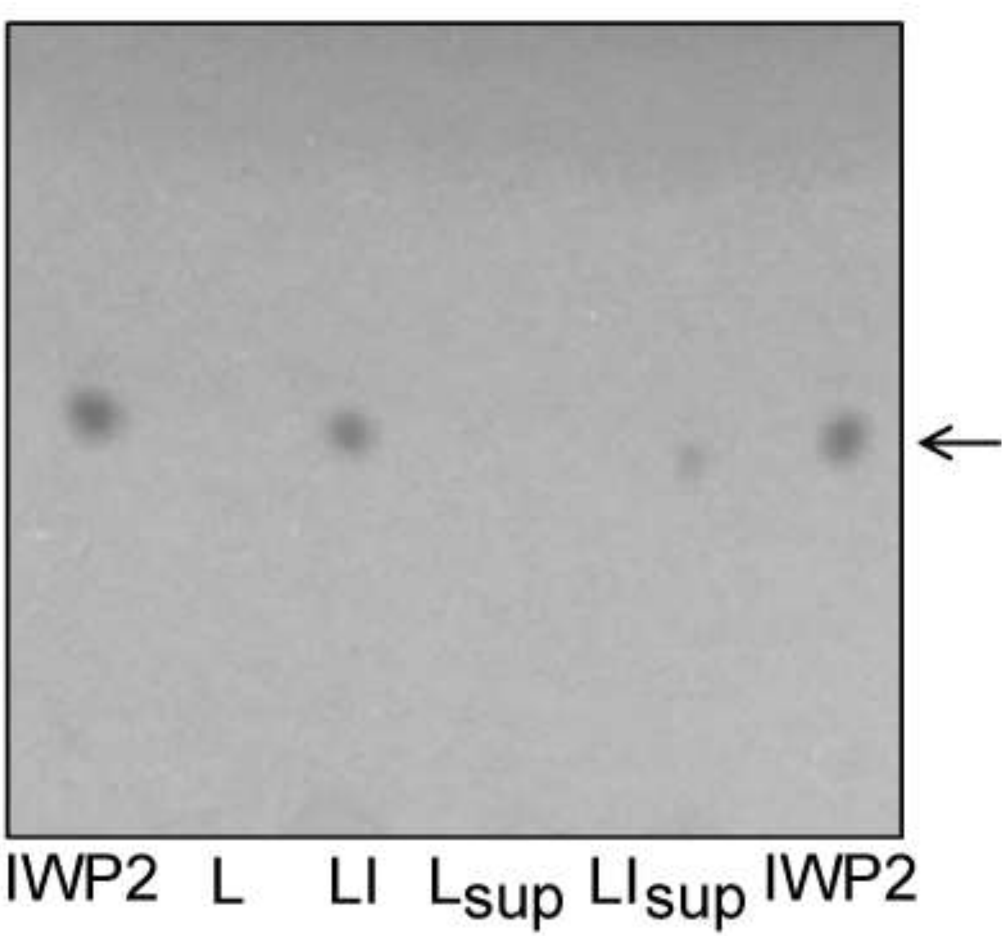

Figure S1. a) Thin Layer Chromatography assessing efficacy of IWP-2 incorporation in Liposomes. Dots corresponding to LI (Liposomal IWP-2) and IWP-2 (free drug) represent extent of liposomal incorporation of IWP-2 (80%) relative to total amount of free drug used (100%). L, LI sup, L sup represent the free liposome (vehicle control), sup collected from LI and L prep respectively.

Figure S1.b

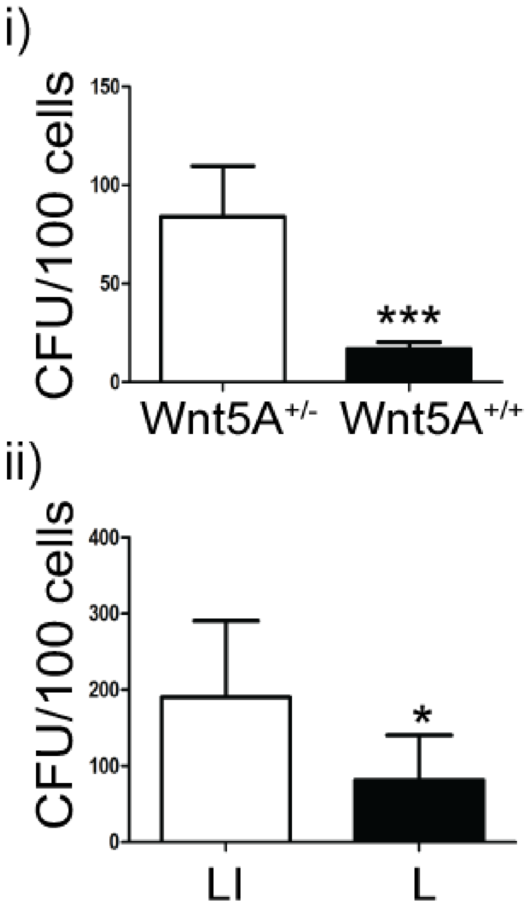

Figure S1.b) Bacterial load increases in peritoneal macrophages upon depletion of Wnt5A. i) CFU/100 cells in peritoneal macrophages harvested from PA challenged Wnt5A $^{+/-}$ and Wnt5A $^{+/+}$ mice. Ii) CFU/100 Cells from PA challenged BALB/c mice pretreated seperately with liposomal formulation of IWP2 (LI) and empty Liposome (L). Data represented as mean ±SEM; significance annotated as follows: ****p*** ≤ 0.05, *****p*** ≤ 0.005, ******p*** ≤ 0.0005.

Figure S1.c

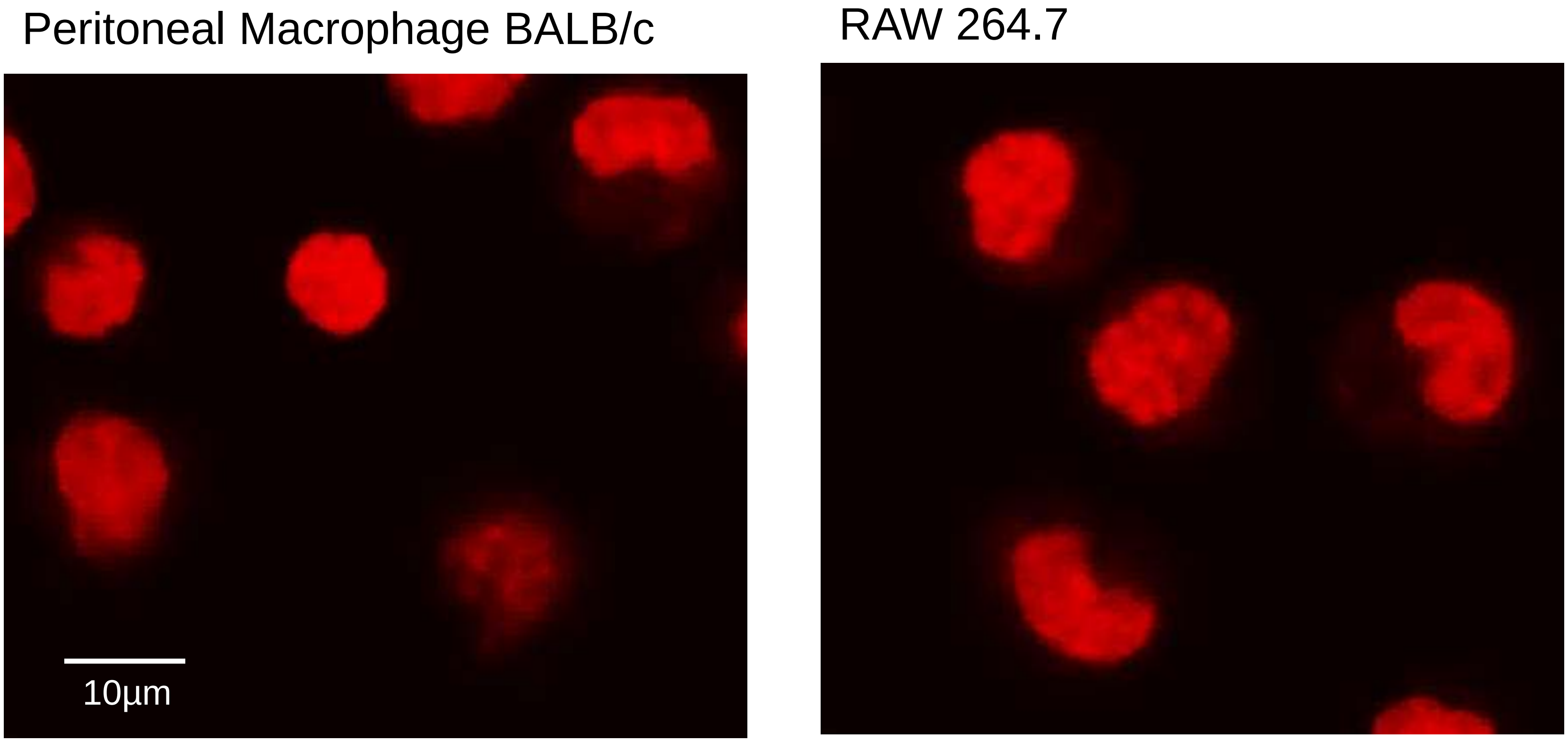

Figure S1.C. PI staining of uninfected macrophages as experimental controls for Fig 1e and 3l

Figure S2

a)

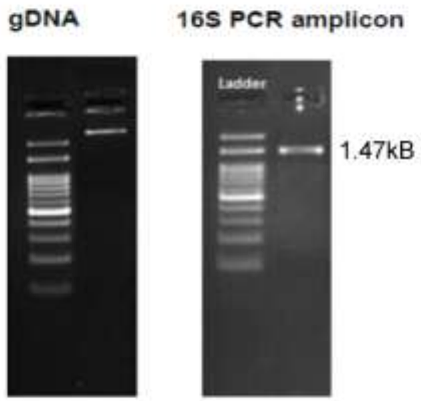

b)

5'TGCTCAGATTGAACGCTGCGGCAGGCCTAACACATGCAAGTCGAGCGGATGAAGGGAGCTTGCTCCTGGATTCAGCGGCGGACGGGT
GAGTAATGCCTAGGAATCTGCCTGGTAGTGGGGGATAACGTCCGGAAACGGGCGCTAATACCGCATACGTCCTGAGGGAGAAAGTGGG
GGATCTTCGGACCTCACGCTATCAGATGAGCCTAGGTCGGATTAGCTAGTTGGTGGGGTAAAGGCCTACCAAGGCGACGATCCGTAACT
GGTCTGAGAGGATGATCAGTCACACTGGAACTGAGACACGGTCCAGACTCCTACGGGAGGCAGCAGTGGGGAATATTGGACAATGGGC
GAAAGCCTGATCCAGCCATGCCGCGTGTGTGAAGAAGGTCTTCGGATTGTAAAGCACTTTAAGTTGGGAGGAAGGGCAGTAAGTTAAT
ACCTTGCTGTTTTGACGTTACCAACAGAATAAGCACCGGCTAACTTCGTGCCAGCAGCCGCGGTAATACGAAGGGTGCAAGCGTTAAT
CGGAATTACTGGGCGTAAAGCGCGCGTAGGTGGTTCAGCAAGTTGGATGTGAAATCCCCGGGCTCAACCTGGGAACTGCATCCAAAAC
TACTGAGCTAGAGTACGGTAGAGGGTGGTGGAATTTCCTGTGTAGCGGTGAAATGCGTAGATATAGGAAGGAACACCAGTGGCGAAGG
CGACCACCTGGACTGATACTGACACTGAGGTGCGAAAGCGTGGGGAGCAAACAGGATTAGATACCCTGGTAGTCCACGCCGTAAACGA
TGTCGACTAGCCGTTGGGATCCTTGAGATCTTAGTGGCGCAGCTAACGCGATAAGTCGACCGCCTGGGGAGTACGGCCGCAAGGTTAA
AACTCAAATGAATTGACGGGGGCCCGCACAAGCGGTGGAGCATGTGGTTTAATTCGAAGCAACGCGAAGAACCTTACCTGGCCTTGAC
ATGCTGAGAACTTTCCAGAGATGGATTGGTGCCTTCGGGAACTCAGACACAGGTGCTGCATGGCTGTCGTCAGCTCGTGTCGTGAGAT
GTTGGGTTAAGTCCCGTAACGAGCGCAACCCTTGTCCTTAGTTACCAGCACCTCGGGTGGGCACTCTAAGGAGACTGCCGGTGACAAA
CCGGAGGAAGGTGGGGATGACGTCAAGTCATCATGGCCCTTACGGCCAGGGCTACACACGTGCTACAATGGTCGGTACAAAGGGTTGC
CAAGCCGCGAGGTGGAGCTAATCCCATAAAACCGATCGTAGTCCGGATCGCAGTCTGCAACTCGACTGCGTGAAGTCGGAATCGCTAG
TAATCGTGAATCAGAATGTCACGGTGAATACGTTCCCGGGCCTTGTACACACCGCCCGTCACACCATGGGAGTGGGTTGCTCCAGAAG
TAGCTAGTCTAACCGCAAGGGGGACGGTTACCACGGAGTGATTCATGACTGGGTGAAGTCGTAACAA 3'

Figure S2. Identification of *P. aeruginosa* isolate. (a) Depiction of the genomic DNA (gDNA) of *P. aeruginosa* isolate. (b) *P. aeruginosa* validated by DNA sequence of 16SrDNA confirming presence of *P. aeruginosa*.

Figure S3

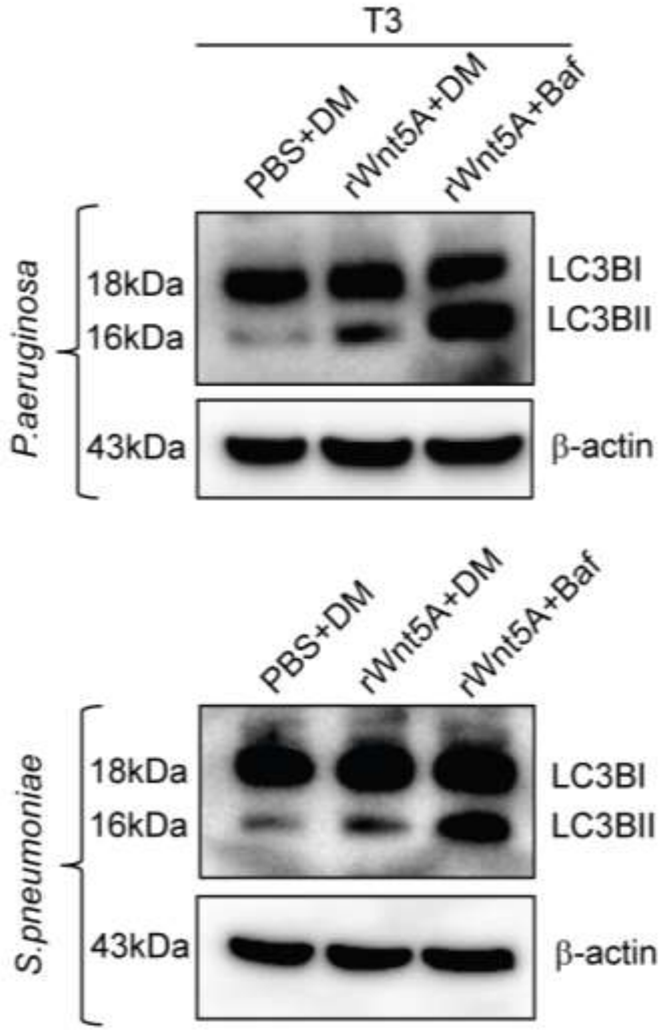

Figure S3. Bafilomycin A1 inhibits autophagic flux increasing Wnt5A induced LC3BII accumulation in *P.aeruginosa* and *S.pneumoniae* infected macrophages. Upper panel; Immunoblot showing increased LC3BII accumulation upon BafilomycinA1 (Baf) treatment in *P.aeuruginosa* infected macrophages. Lower panel; Immunoblot showing increased LC3BII accumulation upon BafilomycinA1 (Baf) treatment in *S.pneumoniae* infected macrophages. T3 denotes 3h post infection by *P.aeruginosa* and *S.pneumoniae*. DM denotes DMSO, vehicle control for Baf.

Figure S4

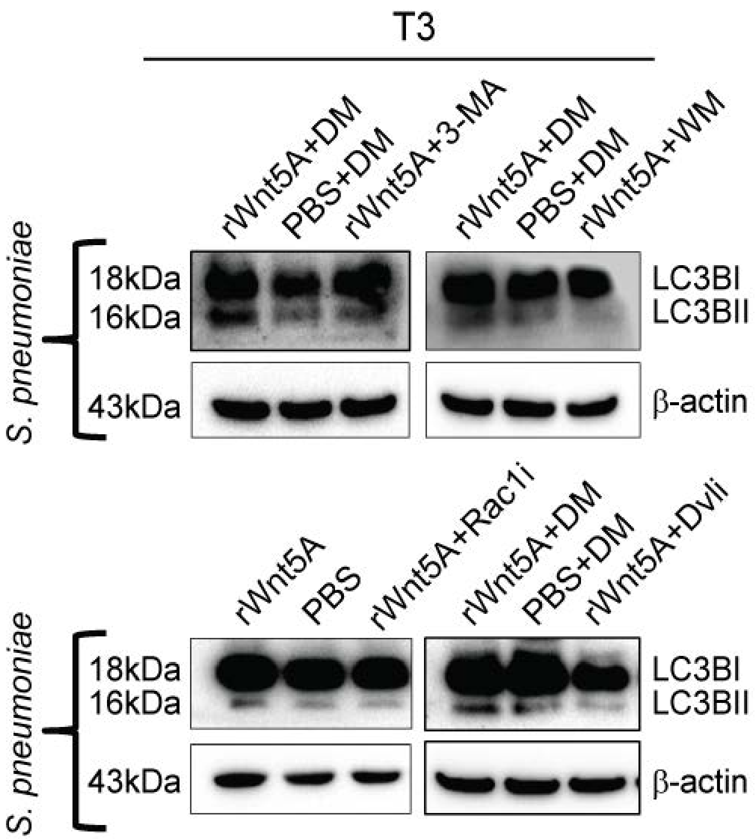

Figure S4. Autophagy inhibitors and Wnt5A signaling inhibitors block LC3BII accumulation in SP infected macrophages pretreated with rWnt5A. Immunoblot showing reduction of LC3BII accumulation 3h (T3) post infection by *S. pneumoniae* in the presence of 3-MA: 3‘methyladenine, WM: Wortmannin, Rac1i: RAC1 inhibior and Dvli: Disheveled inhibitor. DMSO (DM) was used as vehicle control in case of 3-MA, WM and Dvli. PBS was used as vehicle control for rWnt5A and Rac1i.

Figure S5

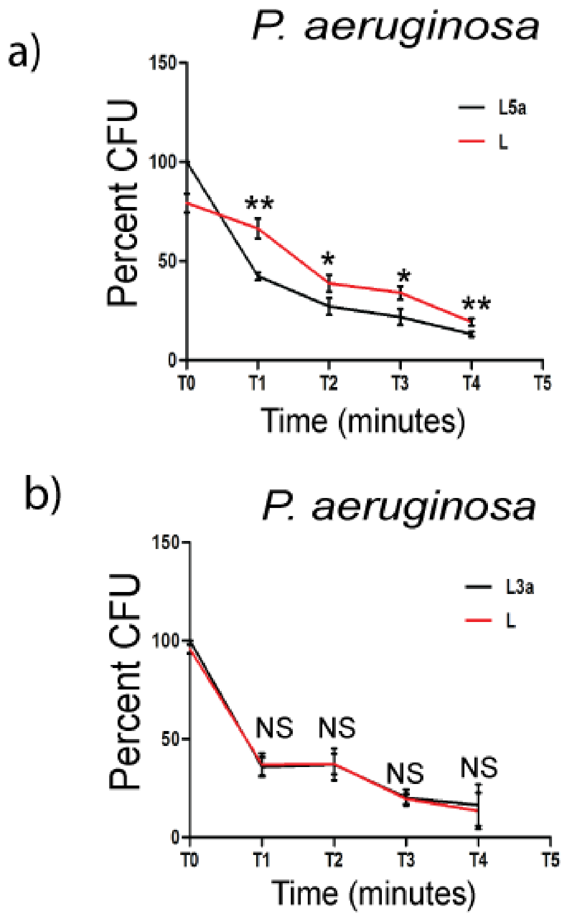

Figure S5. Bacterial killing by Wnt5A usingWnt3A as reference. (a) Effect of L5A (Wnt5A conditioned media) induced bacterial killing from 0h - 4h (T0-T4). (b) L3A (Wnt3A conditioned media) does not have significant effect on bacterial killing at different time points. L (conditioned media derived from L fibroblasts) is used as control for L5A and L3A. Data represented as mean ±SEM; significance annotated as follows: $*p \leq 0.05$, $**p \leq 0.005$, $***p \leq 0.0005$.

Figure S6

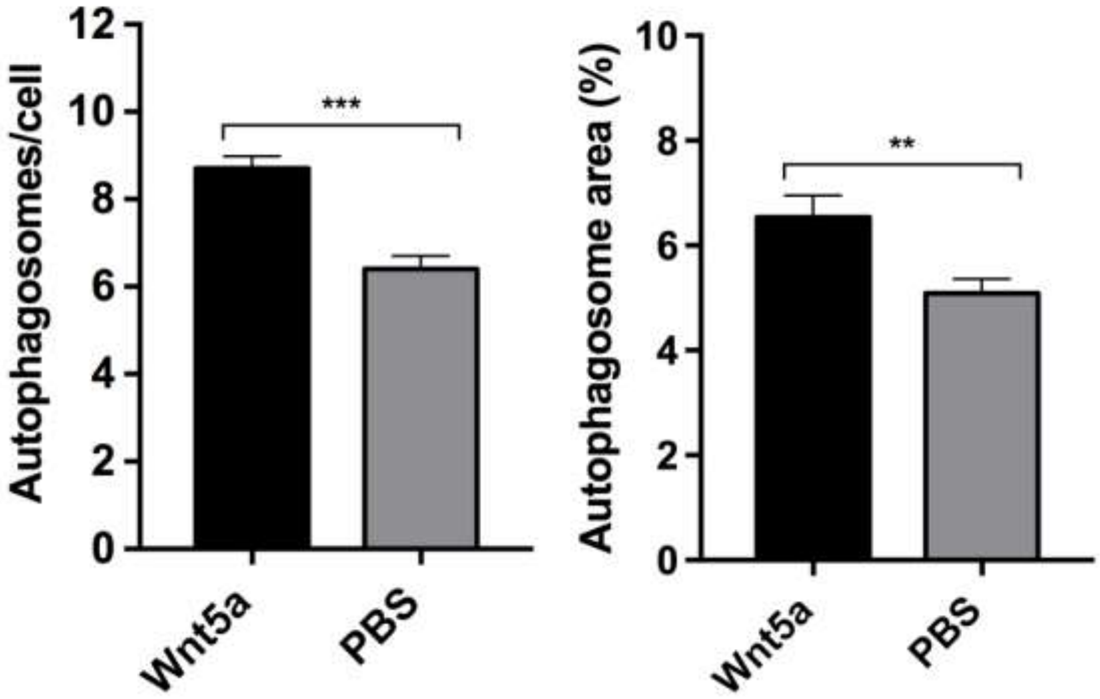

Figure S6. Morphometry of autophagosomes (n=108) by TEM. Data represented as mean ±SEM; significance annotated as follows: **p* ≤ 0.05, ***p* ≤ 0.005, ****p* ≤ 0.0005.

Figure S7

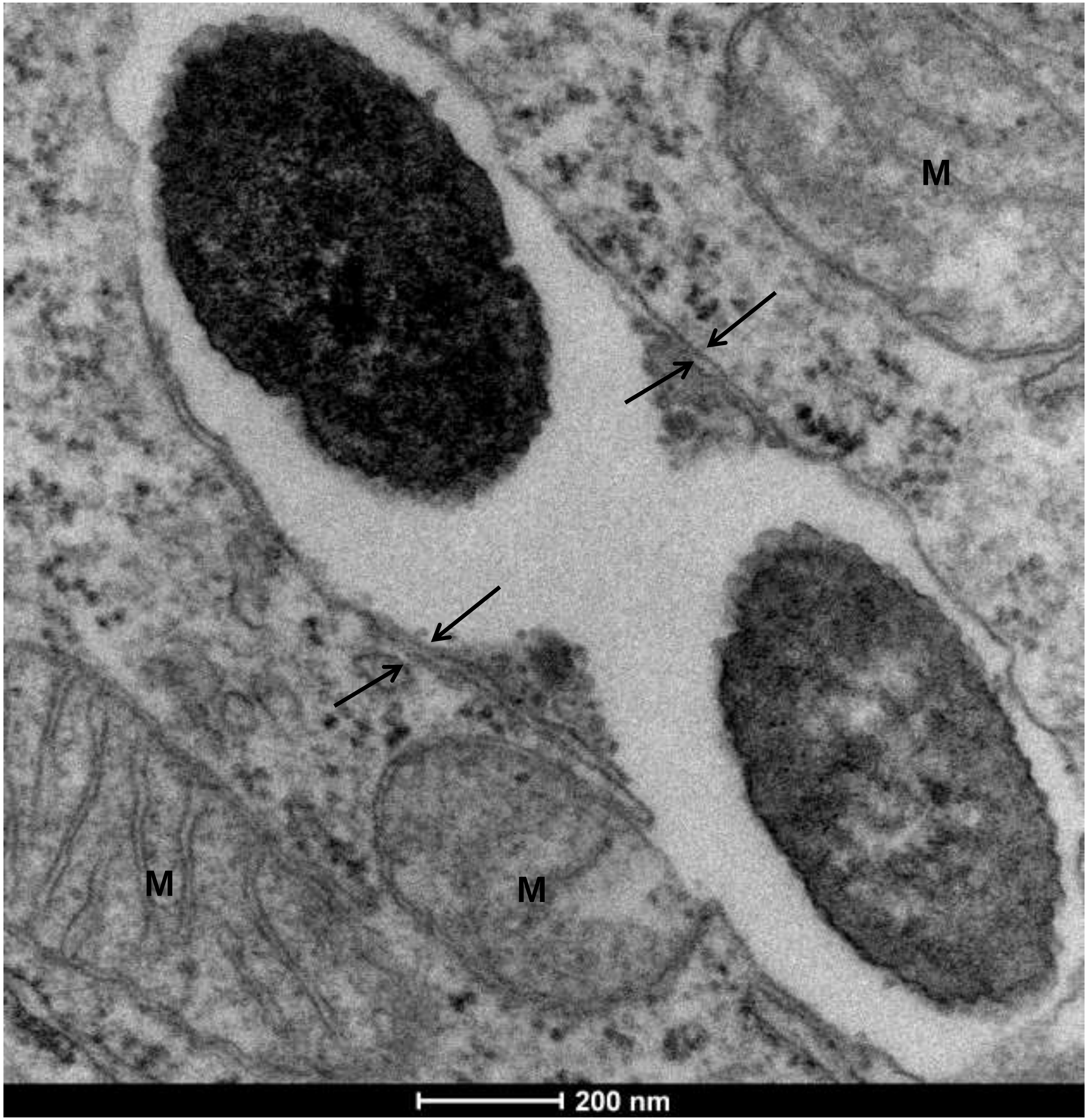

Figure S7. Original version of Figure 8d. Mitochondrial (**M**) fusion with bacteria containing autophagosome. Arrows point to double membrane structures encapsulating bacteria in Wnt5A treated macrophages
